# Identification of a novel missense variant in the *LMX1B* gene associated with nail-patella syndrome in a Chinese family

**DOI:** 10.3389/fgene.2025.1574076

**Published:** 2025-05-12

**Authors:** Qian Sun, Yaqiong Ren, Yue Cao, Wen Zheng, Guanghao Su, Xiaodong Wang, Hongying Wang

**Affiliations:** ^1^ Department of Orthopedics, Children’s Hospital of Soochow University, Suzhou, China; ^2^ Laboratory of Pediatric Research, Children’s Hospital of Wujiang District, Suzhou, China; ^3^ Department of Clinical Laboratory, Children’s Hospital of Soochow University, Suzhou, China

**Keywords:** nail-patella syndrome, LMX1B, whole-exome sequencing, gene variant, musculoskeletal deformity

## Abstract

**Background:**

Nail-patella syndrome (NPS) is an autosomal dominant disorder caused by the variants of the *LMX1B* gene, affecting several systems, including musculoskeletal, renal, and ocular systems. Despite the well-established genetic basis, the complicated relationship between genotype and phenotype still remains unclear. This study aimed to identify the genetic cause of NPS in a Chinese family and elucidate its potential contribution to the disease’s phenotypic spectrum.

**Methods:**

Clinical data and peripheral blood samples were collected from the affected family. Whole-exome sequencing (WES) was conducted to identify potential pathogenic variants, followed by Sanger sequencing to validate the candidate variant. Bioinformatic tools were employed to predict the 3D structure alterations and pathogenicity of the variant. Wild-type and mutant *LMX1B* overexpression plasmids were constructed to investigate the functional consequences of the variant. Western blotting and immunofluorescence were conducted to measure the expression and localization of the protein.

**Results:**

The proband presented with clinical manifestations, including nail malformation, patella dysplasia, restricted elbow movement, and pes planus. Both his mother and sister exhibited symptoms related to the skeletal system. WES identified a novel c.812G>C (p.R271T) variant in the affected family members. Bioinformatic analyses revealed structural modification in the protein and predicted functional impairment. Western blotting showed no significant difference in the expression level between wild-type and mutant protein. However, immunofluorescence demonstrated distinct changes in the subcellular localization of c.812G>C mutant.

**Conclusion:**

NPS is a rare multisystem disorder with variable clinical presentations. In this family, the skeletal system was mainly involved with variations among different members. Our study identified a novel c.812G > c variant in the *LMX1B* gene, changing the nuclear localization of the protein.

## Introduction

Nail-patella syndrome (NPS; OMIM#161200), also known as hereditary osteo-onycho-dysplasia, is a rare autosomal dominant disease characterized by nail hypoplasia, hypoplastic patella, radial head dislocation, and iliac horns, with a reported incidence of 1 in 50,000 (Sweeney, 2003). Regional epidemiological studies have reported a wide variation in prevalence (22 per million in England and 4.5 per million in the United States) (Guidera et al., 1991). The disease predominantly manifests as skeletal abnormalities, ocular complications (particularly glaucoma), and renal dysfunction. However, the involvement of the auditory, neurological, dental, and gastrointestinal systems has also been documented (Bongers E. M. et al., 2005). Despite extensive research, a clear association between genotype and phenotype among patients with NPS remains elusive. Significant phenotypic heterogeneity has been observed at the individual, intrafamilial, and interfamilial levels (Lin et al., 2008).

NPS is caused by heterozygous loss-of-function mutations in the *LMX1B* gene (OMIM#602575), which encodes the LIM-homeodomain transcription factor that plays a critical role in embryonic development. In patients with NPS, *LMX1B* mutations disrupt the normal development of the dorsal-ventral axis in limbs, leading to defects in dorsal structures, such as nails and patella, and causing musculoskeletal symptoms. The variants also affect the expression of collagen IV chains and podocyte differentiation, leading to glomerular basement membrane abnormalities and renal diseases (Chen et al., 1998). These variants lead to altered transcriptional activity and affect the DNA-binding ability of the LMX1B protein, finally resulting in multisystem symptoms in patients with NPS (Dreyer et al., 1998; Dreyer, 2000).

In this study, we reported a Chinese family whose 3 members had NPS, including the proband, his younger sister, and his mother. We also identified a novel c.812G>C missense variant in the *LMX1B* gene, which has never been reported in the literature. We expand the genotypic and phenotypic spectrum of NPS and provide a better understanding of genotype-phenotype correlations in this disease.

## Materials and methods

### Subjects and clinical assessment

This two-generation family from eastern China comprised three affected individuals (Figures 3A, I–2, II-2, and II-2). A thorough assessment of their natural history and physical condition was conducted on all family members. The affected members underwent additional radiological and laboratory examinations. X-ray was conducted to assess their skeletal deformities, while renal function was measured through urinalysis and hematologic tests. Additionally, intraocular pressure measurement using the non-contact tonometer method was conducted. Peripheral blood samples of all members of the family were obtained for further analysis. This study was reviewed and approved by the Institutional Review Board of the Children’s Hospital of Soochow University (approval number: 2024CS056). Written informed consent was obtained from all participants or their legal guardians, authorizing both the standardized collection of clinical records and peripheral blood samples. The subsequent use of genetic testing and scientific dissemination was conducted in compliance with the Declaration of Helsinki.

### Whole-exome sequencing

Whole-exome sequencing (WES) was conducted using the following protocol. Firstly, DNA was extracted from peripheral blood using a Qiagen DNA Blood Midi/Mini kit (Qiagen GmbH, Hilden) following the manufacturer’s protocol. Then, DNA was interrupted to approximately 200bp and then was end-repaired. Subsequently, the DNA fragments were ligated, and fragments with nearly 320bp were collected. After PCR amplification, DNA fragments were hybridized and captured using Berry’s NanoWES Human Exome V1.0 (Berry Genomics) following the manufacturer’s protocol. Next, the libraries were measured using qPCR, and size distribution was determined using an Agilent Bioanalyzer 2,100 (Agilent Technologies). Finally, the Novaseq6000 platform (Illumina), with 150 bp pair-end sequencing mode, was employed for sequencing the captured exome library. The sequencing reads were aligned to the human reference genome (hg38/GRCh38) utilizing Burrows–Wheeler Aligner tool. PCR duplicates were deleted using Picard v1.57 (http://picard.sourceforge.net/). Verita Trekker^®^ Variants Detection System by Berry Genomics and the third-party software GATK (https://software.broadinstitute.org/gatk/) were employed for variant calling. Variant annotation and interpretation were conducted using ANNOVAR (Wang et al., 2010) and the Variants Annotation Interpretation System. Human population databases, such as 1,000 Genome Project (http://browser.1000genomes.org), were employed to exclude high-frequency variants.

Variants with minor allele frequency (MAF) ≥1% in public databases or variants existing in in-house controls were excluded. Only variants in exonic regions or with potential splicing effects were retained. In silico tools, including REVEL, SIFT, Polyphen2, Variant Taster, CADD, and PROVEAN, were employed to predict the pathogenicity of the remaining variants. Disease and phenotype databases, such as OMIM (http://www.omim.org), ClinVar (http://www.ncbi.nlm.nih.gov/clinvar), and HGMD (http://www.hgmd.org), were utilized for further assessment. In trio analysis, the variants were filtered to retain those that fit the expected inheritance pattern for the disease. The candidate variants were then classified following the ACMG guidelines (Richards et al., 2015) to determine their pathogenicity. Thereafter, further bioinformatics analysis and Sanger sequencing were employed.

### Bioinformatics analysis

The three-dimensional structure of the wild-type LMX1B protein was obtained from the AlphaFold Protein Structure Database to analyze the potential functional effect of the identified genetic variants. The three-dimensional conformation of both wild-type and mutant LMX1B proteins and their target DNA sequence FLAT-E (5′-TAATTA-3′) (German et al., 1992) were modeled using AlphaFold 3. Next, molecular docking simulations were conducted using PyDockDNA (Rodríguez-Lumbreras et al., 2022). The predicted structural models and protein-DNA docking complexes were visualized and analyzed using PyMOL (Version 2.5; Schrödinger, LLC). NCBI HomoloGene was employed to analyze the conservation of the variant amino acid residue among different species.

### Sanger sequencing

Sanger sequencing was conducted to validate the c.812 G>C variant identified by WES. Primers were designed using Primer5 using the following sequences: forward: 5′- ACCCACCATCTCCCCGTT -3′ and reverse: 5′- CGC​CAG​CTT​CTT​CAT​CTG​C -3′. Phanta^®^ Max Super-Fidelity DNA Polymerase (Nanjing Novogene Bioinformatics Technology Co., Ltd.) was employed for PCR amplification. Following purification, the PCR product was sequenced using an AB1 3500DX instrument (Applied Biosystems, United States).

### Plasmid construction

The full-length wild-type human *LMX1B* cDNA (NM_001174147.2) was cloned into the C-terminal region of pcDNA3.1-3×Flag (EGFP + PURO) expression vector to generate a recombinant plasmid for overexpression studies. Using a site-directed mutagenesis kit (TIANGEN, KM101, and Beyotime, D0206S), mutagenesis was conducted to introduce the specific point variants c.737G>A and c.812G>C into the *LMX1B*-coding sequence. The following mutagenic primers were utilized: c.737G>A-forward: 5′- CTC​GTC​GAA​GCC​TTG​CCA​AAA​GGT​CCG​AGA-3′, c.737G>A-reverse: 5′-TGG​CAA​GGC​TTC​GAC​GAG​ACC​TCG​AAG​GAG​G-3'; c.812G>C-forward: 5′-CCA​GGT​CTG​GTT​TCA​GAA​CCA​AAC​AGC​AAA​GAT​GAA​GAA​GC-3′, c.812G>C- reverse: 5′-GCT​TCT​TCA​TCT​TTG​CTG​TTT​GGT​TCT​GAA​ACC​AGA​CCT​GG-3'.

### Cell culture and transfection

HEK293T and HeLa cell lines were cultured in Dulbecco’s Modified Eagle Medium (Gibco, C11995500BT) containing 10% fetal bovine serum (Vivacell, C04001), 1% penicillin-streptomycin (Gibco, 15140122), and 1% L-glutamine (Solarbio, G0200). Cells were seeded into culture plates 1 day before transfection. After reaching 60%–70% cell confluence, transfection was conducted using Lipofectamine 3,000 reagent (Invitrogen, L3000015) following the manufacturer’s instructions. Briefly, DNA-lipid complexes were prepared by mixing the appropriate amount of plasmid DNA with Lipofectamine 3,000 reagent in a serum-free medium and incubating the mixture at room temperature for 10–15 min. The transfection complexes were gently added to the cells, followed by incubation under standard culture conditions (37°C, 5% CO_2_). The cells were harvested for protein extraction or immunofluorescence staining 48 h after transfection.

### Western blotting

Total proteins were extracted from HEK293T and HeLa cells using RIPA lysis buffer (Beyotime, P0013B) containing protease inhibitor cocktail (apexbio, K1007) and phosphatase inhibitor cocktail (apexbio, L1015). The concentration of proteins was measured using the BCA protein assay kit (Servicebio, G2026) following the manufacturer’s instructions. Equal amounts of protein (10 μg) were separated using 10% sodium dodecyl sulfate-polyacrylamide gel electrophoresis (SDS-PAGE) and then transferred onto polyvinylidene difluoride (PVDF) membranes (Millipore, IPFL00010). The membranes were blocked with 5% non-fat milk in TBST (TBS containing 0.1% Tween-20) at room temperature for 1 h. Subsequently, the membranes were incubated with primary antibodies against Flag (ABclonal, AE092; 1:10,000) and GAPDH (Cell Signaling Technology, 2118S; 1:6,000) at 4°C overnight. After three washes with TBST, the membranes were incubated with horseradish peroxidase (HRP)-conjugated goat anti-rabbit secondary antibody (ZSGB-BIO, ZB-2301; 1:5,000) at room temperature for 1 h. Protein bands were visualized using enhanced chemiluminescence (ECL) reagent (Servicebio, G2074) and detected using an automated chemiluminescence imaging system (Tannon, 4,160).

### Immunofluorescence

The cells were washed three times with phosphate-buffered saline (PBS) at room temperature. Subsequently, the cells were fixed with 4% paraformaldehyde (Sigma-Aldrich, P6148) at room temperature for 15 min, followed by three rounds of 5-minute washes with PBS. The cells were blocked with the blocking buffer containing 5% normal goat serum (Jackson ImmunoResearch, 005–000–121) and 0.3% Triton X-100 (biosharp, BS084) in PBS at room temperature for 1 h to minimize non-specific binding. After blocking, the cells were incubated with primary anti-Flag antibody (ABclonal, AE092; 1:200) at 4°C overnight. The next day, the cells were washed three times with PBST (PBS containing 0.02% Tween-20) and three times with PBS, with each wash lasting 5 min. Then, the cells were dark incubated with CoraLite488-conjugated goat anti-rabbit secondary antibody (Proteintech, SA00013-2) at room temperature for 1 h. Nuclear staining was conducted using DAPI (Servicebio, G1012) for 10 min. Finally, the slides were mounted using an anti-fade mounting medium (Yeasen, 36307ES08). Under consistent imaging parameters, all images were captured using a confocal microscope (ZEISS, LSM 800).

## Results

### Clinical assessment revealed that the three affected members in this family mainly exhibited musculoskeletal malformations

**FIGURE 1:**
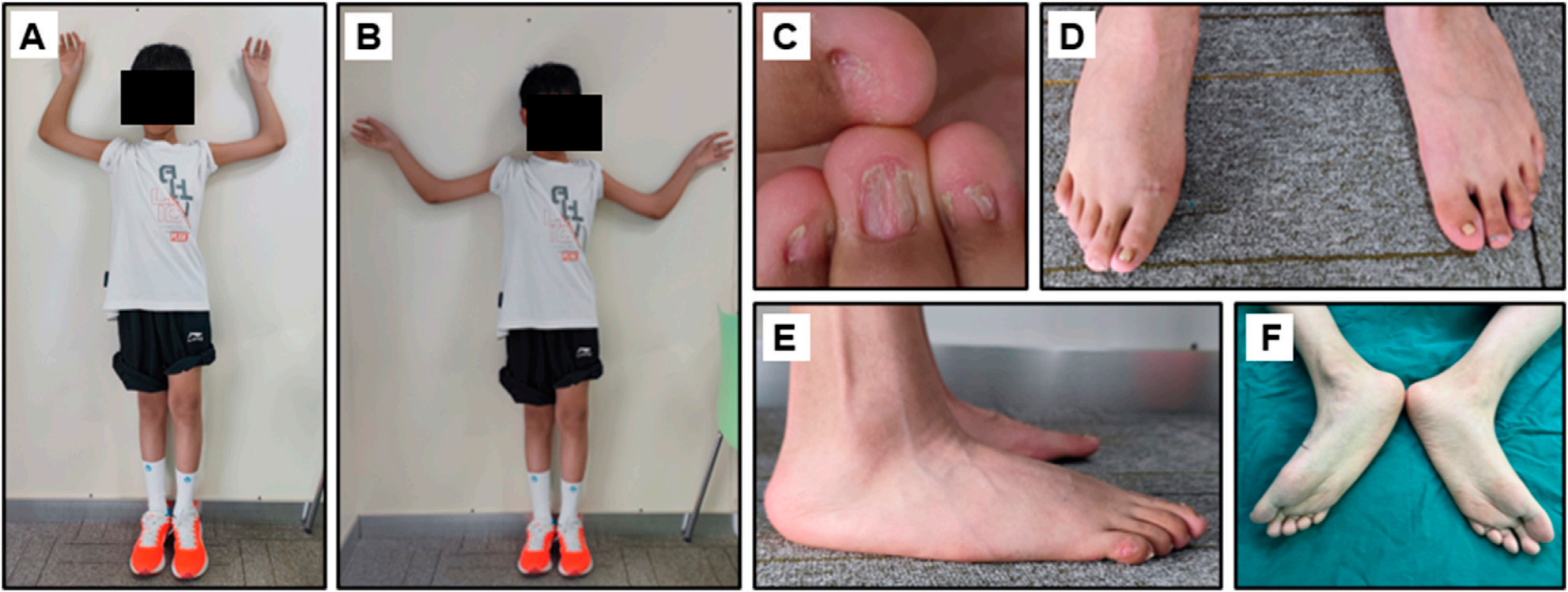
Clinical manifestation of the proband. **(A, B)** Limited range of motion of the elbows and the hypoplastic patella. **(C)** Dysplastic fingernails. **(D**–**F)** Hypoplastic thumbnails of the feet and the Pes planus of the feet.

**FIGURE 2:**
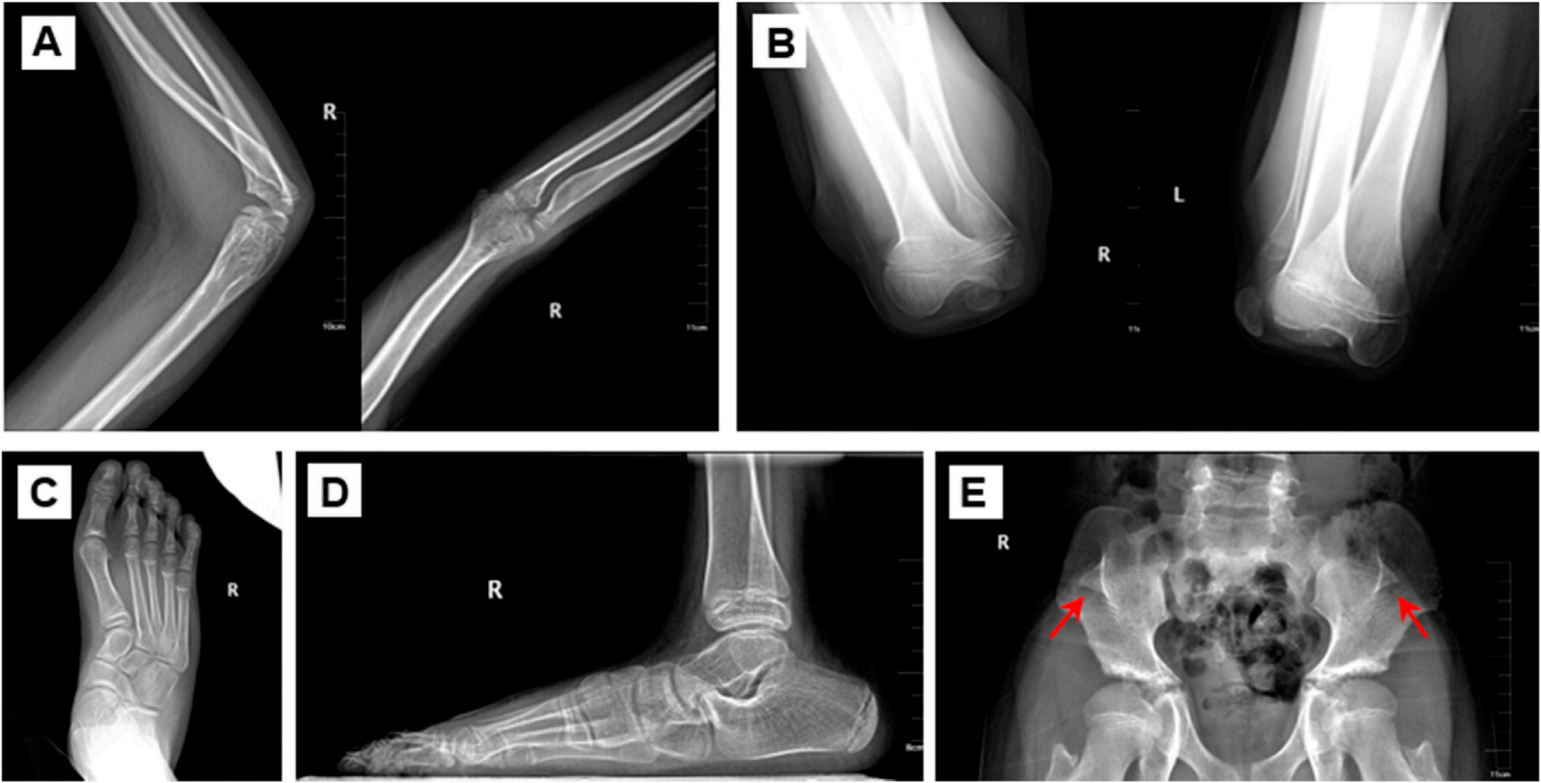
Radiological assessment of the proband. **(A)** Dislocated radial head **(B)** Bilateral patellar dysplasia and displacement. **(C, D)** Pes planus of the feet. **(E)** Iliac horns. The arrow refers to the iliac horns.

The proband was an eleven-year-old boy with bilateral elbow dysplasia, nail deformities, and a right flat foot. He suffered from bilateral flexion contractures of the elbows, with complete loss of pronation and supination. The range of motion of both elbows was severely limited, with only approximately 40° of mobility. Antecubital pterygia was also observed (Figures 1A, B). Nail bed shortening and longitudinal ridging were observed on the hands, particularly on the second to fourth fingers (Figure 1C). The lunula of all nails was absent. The first toenails on both feet were hypoplastic, while the morphology of the remaining toenails was relatively normal (Figure 1D). Pes planus of the right foot was also observed (Figures 1E, F). Radiographic images demonstrated dislocation and hypoplasia of the radial heads (Figure 2A). Additionally, bilateral patellar hypoplasia and dislocation were observed; however, the proband was asymptomatic (Figure 2B). Consistent with pes planus, radiographic examination revealed eversion of the first metatarsal bone in the right foot (Figures 2C, D). Notably, iliac horns, a characteristic feature of NPS, were evident on radiographic examination (Figure 2E).

The proband’s mother, a 38-year-old woman presented with milder manifestations. Physical examination exhibited mildly restricted flexion, extension, pronation, and supination of both elbows (Supplementary Figure S1A). Additionally, hypoplasia of the nails was limited to bilateral thumbnails. Notably, there was a loss of creases in the skin overlying the dorsal surface of the distal interphalangeal joints (Supplementary Figure S1B). The proband’s 4-month-old sister was also affected, exhibiting flexion contractures of the bilateral elbows and nail hypoplasia. Her nails displayed typical triangular lunulae and longitudinal ridging (Supplementary Figure S1C). Physical examination did not reveal pes planus or patellar dysplasia in this infant. Subluxation of the right radial head and dislocation of the left radial head were evident on radiographic imaging (Supplementary Figure S2A). Bilateral hypoplastic patella was also identified on imaging, without patellar dislocations (Supplementary Figure S2B). In radiological assessment, iliac horns were visible only on the right side. Furthermore, bilateral acetabular dysplasia was detected, with more extensive involvement on the left side (Supplementary Figure S2C).

Renal dysfunction was not detected in any of the affected family members. Similarly, glaucoma and ocular hypertension were not detected (Supplementary Table S1). The clinical manifestations of the patients are summarized in Table 1.

**TABLE 1:** Clinical manifestations of the affected members.

Clinical Manifestations	Proband	Mother	Sister
Age	11 years	38 years	6 months
Sex	Male	Female	Female
Nail deformities	Present	Present	Present
Elbow dysplasia	Present (bilateral)	Present (bilateral)	Present (bilateral)
Hypoplastic patella	Present (bilateral)	Present (bilateral)	N/A
Pes planus	Present (right foot)	Absent	Absent
Iliac horns	Present	Present	N/A
Renal dysfunction	Absent	Absent	Absent
Glaucoma	Absent	Absent	Absent
Ocular hypertension	Absent	Absent	Absent

“N/A″ indicates that the examination was not conducted.

### Genetic analysis identified a novel c.812 G>C missense variant in the *LMX1B* gene

**FIGURE 3:**
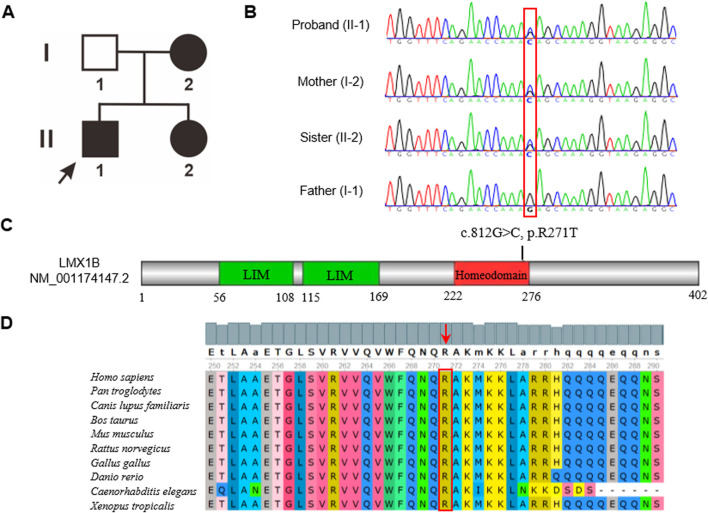
**(A)** The pedigree of this family. Patients are indicated by solid black. The black arrow shows the proband. **(B)** Sanger sequence analysis of the family. Variant c.812G>C (p.R271T) was found in the affected members. While the proband’s father had the wild-type variant. **(C)** Schematic representation of *LMX1B*. The variant in our study was located in the homeodomain. **(D)** The amino acid in the 271 position was highly conserved among different species.

The two-generation pedigree of the family with NPS comprised three affected individuals, with an autosomal recessive inheritance pattern (Figure 3A). WES revealed a heterozygous c.812G>C (p.R271T) variant in exon 5 of the *LMX1B* gene (NM_001174147.2), segregated in all three affected family members. The variant was absent in the proband’s unaffected father, which was confirmed by Sanger sequencing (Figure 3B). This variant was absent in major population databases (Exome Aggregation Consortium and 1000 Genomes Project) and the HGMD database. Computational pathogenicity analysis using REVEL and other five prediction algorithms unanimously classified the c.812G>C variant as a deleterious variant (Table 2). Additionally, this variant was classified as “Likely Pathogenic” based on the 2015 ACMG/AMP guideline (Richards et al., 2015). The c.812G>C variant localized to the homeodomain (HD) of the *LMX1B* gene (Figure 3C), a conserved DNA-binding domain involved in transcriptional regulation. Conservation analysis across 10 representative vertebrate species revealed complete preservation of the arginine residue at position 271 (Figure 3D).

**TABLE 2:** Bioinformatics predictions of pathogenicity for variant p.R271T. All analyses indicated that the variant is pathogenic.

Tool	REVEL	SIFT	Polyphen2	Mutation taster	CADD	PROVEAN
Prediction	Damaging (0.98)	Damaging	Probably damaging	Disease causing	Damaging (28.0)	Damaging

**FIGURE 4:**
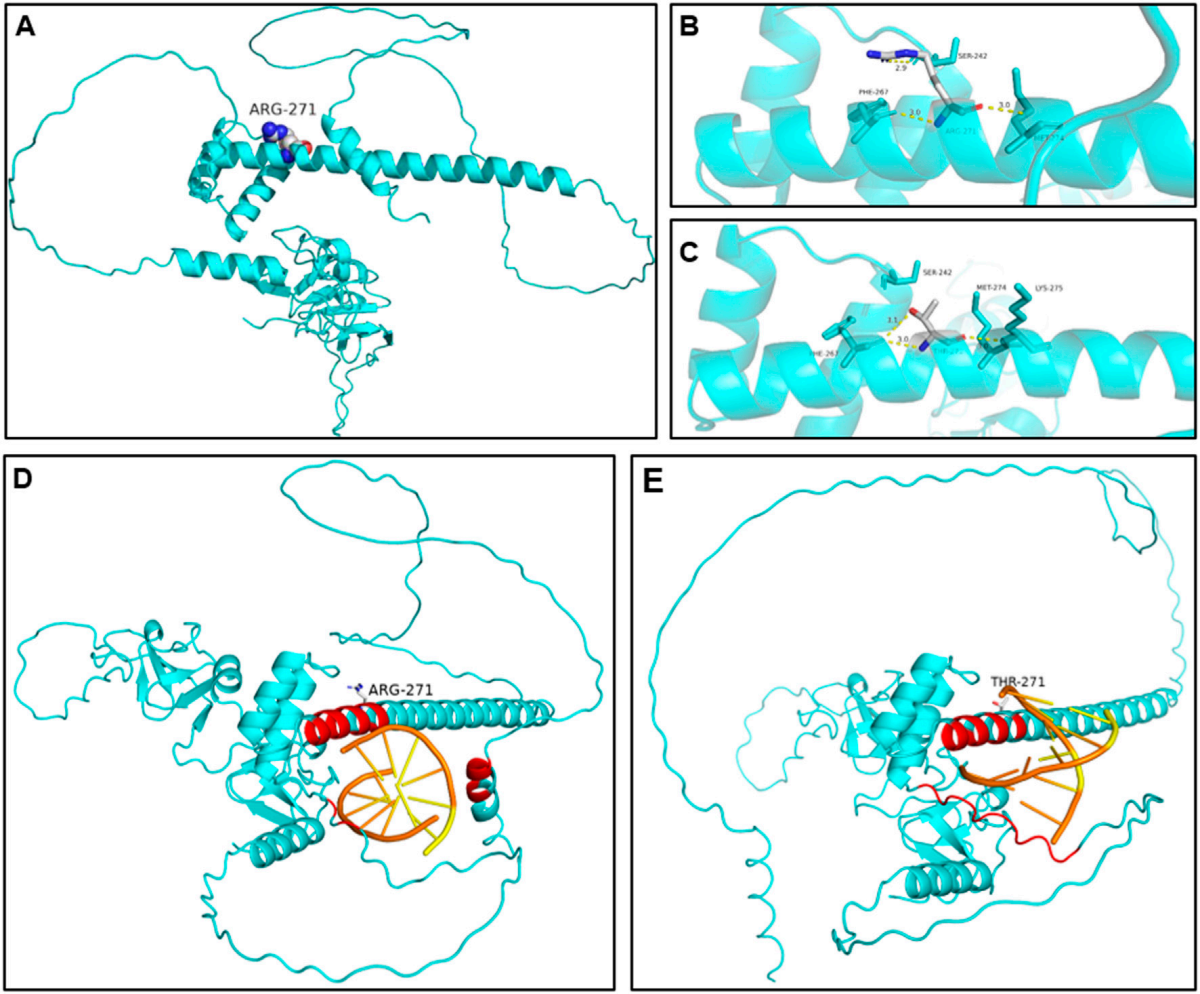
The predicted structural alterations of the mutated protein. **(A, B)** 3D structure of the wild-type LMX1B protein. **(C)** 3D structure of the variant type protein. Hydrogen bonds between positions 271 and 242 and 274 were disrupted. New hydrogen bonds were formed between positions 271 and 267 and 275. The rearrangement of the hydrogen disturbed the structure of the homeodomain, impairing its ability to bind to DNA. **(D)** Molecular docking simulation of wild-type LMX1B-DNA. Effective interaction pairs involve amino acid residues 261–269 and 271–273. **(E)** Molecular docking simulation of variant LMX1B-DNA. Effective interaction pairs involve amino acid residues 261–266, 268–269, and 272. Effective interaction pairs are labeled as red for amino acid residues and as orange for DNA nucleotides.

Structural modeling identified hydrogen-bond network rearrangement and DNA-binding interface destabilization in the *LMX1B* c.812G>C variant. Structural modeling employing AlphaFold (AF-O60663-F1-v4) indicated critical hydrogen-bonding interactions involved in protein stability. In wild-type LMX1B, Arg^271^ formed three essential bonds: a 2.9Å bond with Glu^242^ and two 3.0Å bonds with Asp^267^ and Thr^274^ (Figures 4A, B). The c.812G>C variant disrupted this network, eliminating the Glu^242^ and Thr^274^ interactions while forming aberrant 3.01Å and 3.0Å bonds respectively between the mutant threonine and Leu^267^ and Val^275^ (Figure 4C), resulting in helical structure changes in the homeodomain.

We utilized PyDockDNA to simulate the protein-DNA docking interactions. Figure 4D and E depict the results of the docking simulation. The structure in the protein-DNA binding region with an atom distance less than 7Å was recognized as effective interacting pairs (Baek et al., 2024), labeled as red on the protein and orange on the DNA. For wild-type LMX1B, the effective interface region consisted of amino acid residues 261–269 and 271–273. The variant protein exhibited a distinct interaction pattern, involving residues 261–266, 268–269, and 272 at the binding interface. The direction and binding position of the DNA site was altered in the variant. Although the overall PyDockDNA binding scores were not significantly different between the wild-type and mutant proteins (−97.692 vs. −100.056, Table 3), the variant LMX1B exhibited enhanced van der Waals forces (−21.801 for wild-type and 14.236 for the mutant variant), suggesting impaired binding functionality.

**TABLE 3:** PyDockDNA simulation of the wild-type and variant LMX1B protein-DNA docking.

Sturcture	Configuration	Electrostatics	Van der waals	PyDockDNA (no desolv)
Wild-type	4,603	−95.512	−21.801	−97.692
Variant	636	−101.480	14.236	−100.056

### *In vitro* assays revealed altered subcellular localization caused by the *LMX1B* c.812G>C variant

**FIGURE 5:**
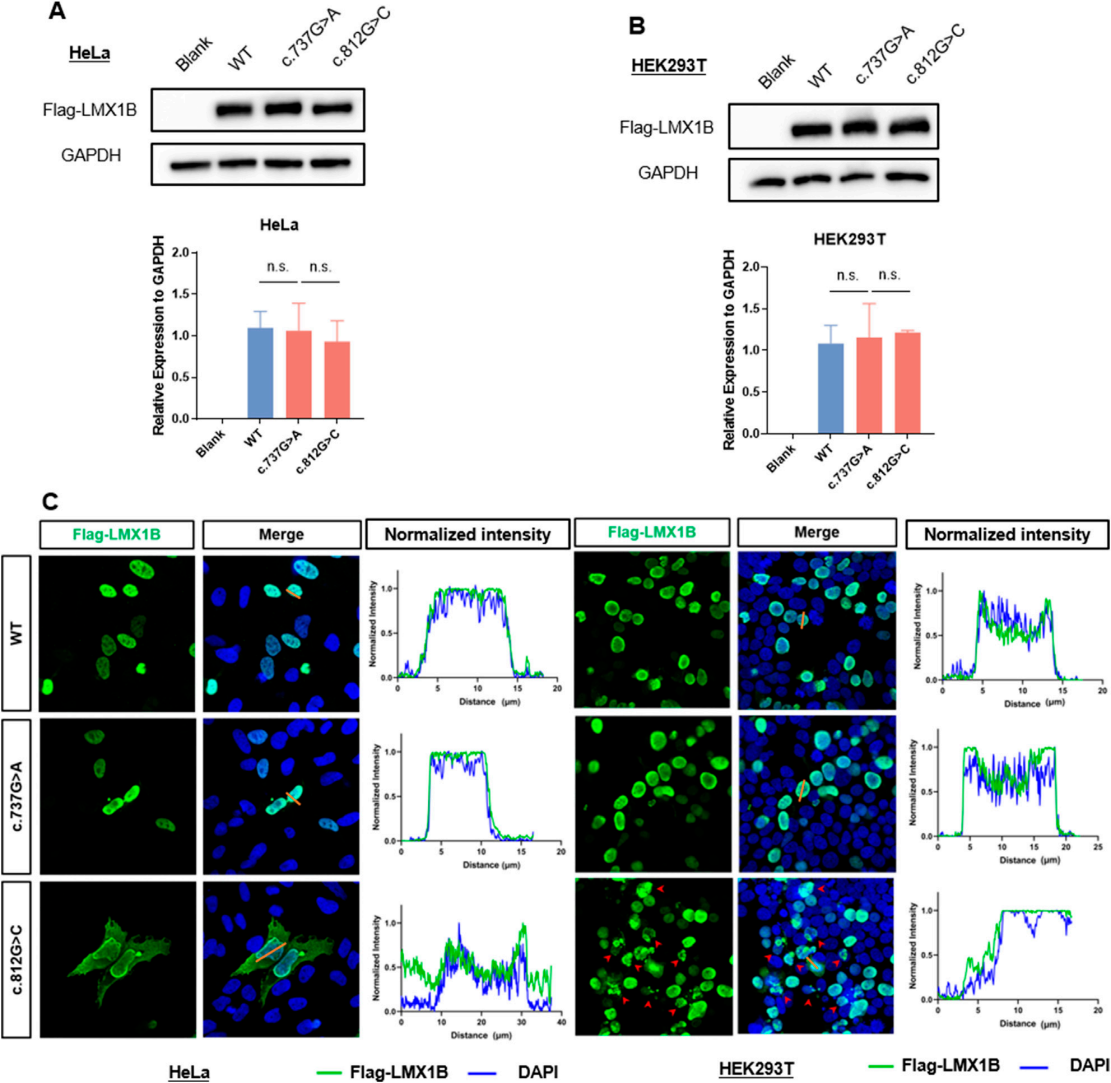
*In vitro* experiment analysis the expression level and subcelluar distribution of variation. **(A)** The protein expression level of LMX1B in HeLa cells overexpressing wild-type *LMX1B*, c.737G>A variant, and c.812G>C variant. **(B)** The protein expression level of LMX1B in HEK293T cells overexpressing wild-type *LMX1B*, c.737G>A variant, and c.812G>C variant. **(C)** Immunofluorescence staining showed subcellular localization of LMX1B variants in HeLa and HEK293T cells. In HeLa cells, the wild-type and c.737G>A mutant proteins were predominantly localized in the nucleus, whereas the c.812G>C mutant exhibited aberrant cytoplasmic distribution. In HEK293T cells, the wild-type and c.737G>A mutant proteins also showed nuclear localization, but the c.812G>C mutant induced pathological nuclear aggregation (indicated by red arrows), suggesting a cell type-specific mislocalization pattern. The normalized fluorescence intensities were quantified on the right side of the figure. The corresponding normalized fluorescence intensities of the straight orange lines in are displayed on the right side of merge image.

Western blotting and immunofluorescence were conducted to measure the expression and localization of wild-type and mutant LMX1B protein and investigate the pathogenicity of the c.812G>C variant. The c.737G>A variant, a known pathogenic variant (Andeen et al., 2017), was employed as a positive control. Western blotting revealed no significant difference in the expression of mutant proteins compared to the wild-type protein (Figures 5A, B). Compared to the wild-type protein and c.737G>A mutant protein, which showed the nuclear localization of LMX1B, immunofluorescence staining revealed a cell type-specific mislocalization of the c.812G>C mutant. This variant induced aberrant cytoplasmic distribution of LMX1B in HeLa cells, but in HEK293T cells, it induced pathological nuclear aggregation, suggesting the potential pathogenic effects of this variant by disrupting normal protein localization (Figure 5C).

## Discussion

NPS is a complex hereditary disorder affecting multiple systems. It is primarily characterized by nail and patellar abnormalities and renal dysfunction (Harita et al., 2016). Additionally, the ocular (Pallozzi Lavorante et al., 2022), nervous, dental, and digestive systems can simultaneously exist (Ghoumid et al., 2015; Witzgall, 2017). In this study, the patients carried the *LMX1B* c.812G>C variant and mainly suffered from musculoskeletal symptoms, including elbow malformation, nail deformities, and patella dysplasia.

The LMX1B protein contains two zinc-binding LIM domains at the NH2-terminus and a DNA-binding homeodomain. The LIM domains are responsible for protein-protein interactions (Kadrmas and Beckerle, 2004). The homeodomain is a highly conserved domain constituting a sequence-specific DNA-binding motif involved in the modulation of gene transcription. More than 200 pathogenic variants of *LMX1B* have been recognized so far (Castilla-Ibeas et al., 2024).

Variants located in the LIM domain are primarily associated with abnormal protein-protein interactions, whereas variants in the homeodomain predominantly impair protein-DNA binding. Some studies have suggested that homeodomain variants may be associated with renal dysfunction (Bongers E. M. et al., 2005). Some variants located in the non-coding region of the *LMX1B* gene only lead to musculoskeletal symptoms (Haro et al., 2021). Apart from the above-mentioned examples, no clear association has been identified between mutation sites and patient phenotypes.

German et al. identified FLAT-E and FLAT-F as the binding sites of the homeodomain (German et al., 1992). Sandra et al. (Dreyer et al., 2000) also confirmed that LMX1B protein regulates transcription by binding to these sequences, and loss-of-function variants in *LMX1B* can lead to the development of NPS. In our study, AlphaFold simulations revealed structural alterations in the helical conformation and hydrogen bonding patterns within the homeodomain. Molecular docking simulations visualized the protein-DNA effective interacting pair modification, indicating pathogenic changes in protein structure. The similarity in binding scores between wild-type and variant proteins can be attributed to two key factors. First, molecular simulations predominantly analyze structural and spatial interactions but do not assess biological function. Second, parameter constraints in these models may not reproduce physiologically relevant binding dynamics. However, based on other functional predictions and the higher van der Waals forces, we believe that the predictive model still supports the pathogenicity of the mutation.

Due to its well-established pathogenicity, proximity to the mutation site (both were located within the homeodomain), and shared pathogenic mechanisms (impaired DNA-binding activity), the c.737G>A variant was selected as a positive control in our *in vitro* experiments. This alignment ensures comparability in assessing the functional effects of homeodomain disruptions.

The cell type-dependent mislocalization patterns of the *LMX1B* c.812G>C variant suggests a previously unrecognized layer of complexity in nuclear protein homeostasis. While HeLa cells exhibited nucleocytoplasmic redistribution, the aberrant nuclear aggregation in HEK293T cells suggests distinct and cell-specific pathogenic mechanisms. This dichotomy may be due to the differential expression of nuclear transport chaperones between epithelial-derived HeLa and embryonic kidney-derived 293T cells. As a transcription factor, LMX1B typically shuttles between the cytoplasm and nucleus in physiological conditions. The wild-type protein binds to DNA to regulate gene transcription (Zhou and Qin, 2012). However, the variant protein displayed decreased DNA-binding capacity, resulting in impaired subcellular distribution and functional activity. This observation aligns with the findings of Wang et al. (2014), who reported a family with NPS carrying the *LMX1B*-R198X variant. Similarly, in that family, complete deletion of the DNA-binding domain led to abnormal cytoplasmic-nuclear protein localization. In their study, immunofluorescence analysis confirmed this mislocalization. Furthermore, studies employing electrophoretic mobility shift assay and luciferase reporter assays have demonstrated that pathogenic variants in the homeodomain can impair the DNA-binding capacity and transcriptional activity of LMX1B, confirming functional disruptions at the molecular level (Sato et al., 2005). These findings collectively validate the bioinformatic predictions of pathogenicity for this variant.

NPS is an autosomal dominant disorder primarily caused by haploinsufficiency. Affected individuals are all heterozygous, and no homozygous variants have been reported in humans so far, suggesting that complete loss of *LMX1B* may be embryonically lethal. In contrast, homozygous *Lmx1b* knockout mice (*Lmx1b* ^−/−^) exhibited severe phenotypes, while heterozygous mice (*Lmx1b* ^+/−^) did not show overt manifestations of NPS (Chen et al., 1998). Notably, *Lmx1b* ^+/−^ mice demonstrated decreased compensatory kidney growth after unilateral nephrectomy compared to wild-type mice (*Lmx1b* ^+/+^) (Endele et al., 2007). These findings underscore the critical role of *LMX1B* dosage in normal renal development and suggest that NPS phenotypes originate from insufficient functional LMX1B protein. However, despite all NPS patients being heterozygous and the observed uncertainty in genotype-phenotype correlations, the association between the protein levels of LMX1B and specific clinical manifestations remains unclear.

A distinct genotype-phenotype correlation could not be established in patients with NPS. Even significant phenotypic heterogeneity is observed within the same family. The role of *LMX1B* in limb development is highly complex, involves several downstream genes, and is regulated by various transcription factors. Notably, approximately 10% of patients with NPS do not exhibit variants in the *LMX1B* exons (Bongers E. M. H. F. et al., 2005). Therefore, establishing a clear genotype-phenotype relationship remains challenging. However, our patients primarily presented with skeletal manifestations and normal kidney function. This suggests that in our patients, *LMX1B* dysfunction in regulating limb development was the predominant cause of the observed clinical features.

*LMX1B* is essential for dorsal-ventral patterning during limb development (Vogel et al., 1995), which possibly leads to musculoskeletal symptoms. *LMX1B* is responsible for establishing dorsal identity in the limb mesoderm, where its expression is induced by the dorsal ectodermal signal Wnt7a (Riddle et al., 1995) The expression pattern of *LMX1B* in undifferentiated dorsal mesoderm remains uniform across the tissue and is typically considered an all-or-none phenomenon. This expression pattern implies that *LMX1B* is activated through a threshold mechanism, where a critical level of dorsal ectodermal signaling triggers its induction. Loss of *Lmx1b* in mice can lead to defective patterning of dorsal autopod (hand/foot) and zeugopod (forearm/leg). Autopods show symmetrical ventral characteristics, including dorsal-type footpads, absent dorsal hair follicles and nails, and matching ventral-type muscles, tendons, and ligaments. Variants in *LMX1B* impair dorsalization during limb development, leading to the hypoplasia of dorsal structures, such as nails and patella. Our patients all exhibited elbow joint malformation. The proband also had antecubital pterygium, which refers to skin webbing across the anterior elbow. Given the restricted joint mobility, surgical interventions, such as soft tissue release and radial head excision, are planned to address functional limitations. Radiographic imaging was not conducted due to the young age of the proband’s sister. Given the infant’s developmental immaturity, longitudinal monitoring is essential to observe potential manifestations that may emerge with age. We also identified hip dysplasia in the proband’s mother, a relatively rare finding among patients with NPS (Jacofsky et al., 2003). The mechanism by which *LMX1B* affects hip joint development remains unclear, but it is well-known that *LMX1B* plays a crucial role in skeletal development. Although direct evidence is lacking, hip dysplasia seems to be a phenotype of NPS rather than being two separate conditions manifesting coincidentally.

Identification of potential genotype-phenotype correlations represents a pivotal research direction in NPS. Further studies are needed to illuminate the upstream regulators and downstream effectors affecting the function of *LMX1B*. For instance, Haro et al. identified two *LMX1B*-associated regulatory modules, namely, LARM1 and LARM2, which bind to LMX1B to potentiate its expression during limb dorsalization in discrete spatial patterns. Ablation of these elements exclusively disrupted dorsalization and did not affect other *LMX1B*-mediated processes (Lemley, 2009; Haro et al., 2021). Other studies have delineated that E47 upregulates the transcriptional activity of *LMX1B* (Dreyer, 2000). LDB1 downregulates the activity of *LMX1B* through E47. As a transcription factor involved in different physiological pathways, substantial studies are needed to unravel the complex roles of *LMX1B* and the pathogenesis of NPS.

## Conclusion

In this article, we presented a family with NPS. Through genetic testing, we identified a novel missense variant of the *LMX1B* gene, c.812G>C (p.R271T), located on the exon 5. Further bioinformatic analysis and *in vitro* experiments unveiled the structural modification and abnormal distribution of the mutated protein and its pathogenic effects.

## Data Availability

The datasets presented in this study can be found in online repositories. The names of the repository/repositories and accession number(s) can be found below: https://db.cngb.org/search/project/CNP0005417/, CNP0005417.

## Abbreviations

NPS, Nail-patella syndrome; *LMX1B,* LIM homeobox transcription factor 1 beta; HD, Homeodomain; WES, Whole-exome sequencing; HGMD, Human gene mutation database; PCR, Polymerase chain reaction.
