# Supplementary material for: Identification of a novel missense variant in the LMX1B gene associated with nail-patella syndrome in a Chinese family
Source: Front Genet. 2025 May 12;16:1574076. doi: 10.3389/fgene.2025.1574076 (PMC12104052; doi:10.3389/fgene.2025.1574076)
Supplement: Supplementary file 1 [file DataSheet1.docx]

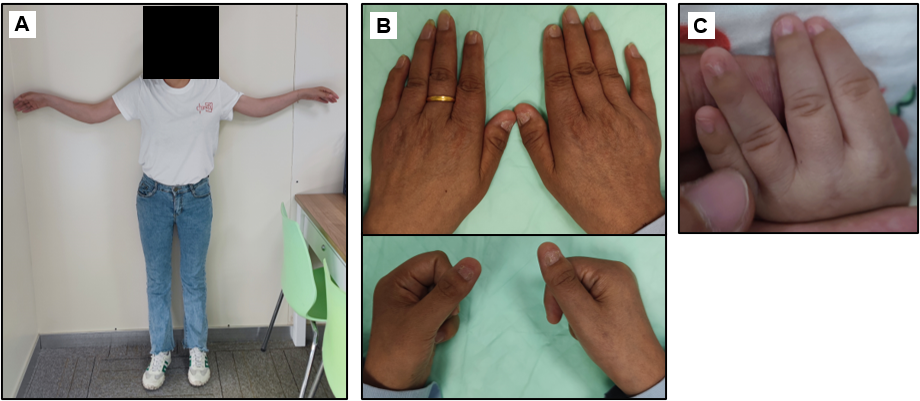


Figure S1: Clinical manifestations in the proband's family members. A: Limited range of motion in the elbows of the proband's mother. B: Nail hypoplasia and absence of skin creases overlying the dorsal aspect of the distal interphalangeal joints in the proband's mother. C: Nail dysplasia in the proband's sister.


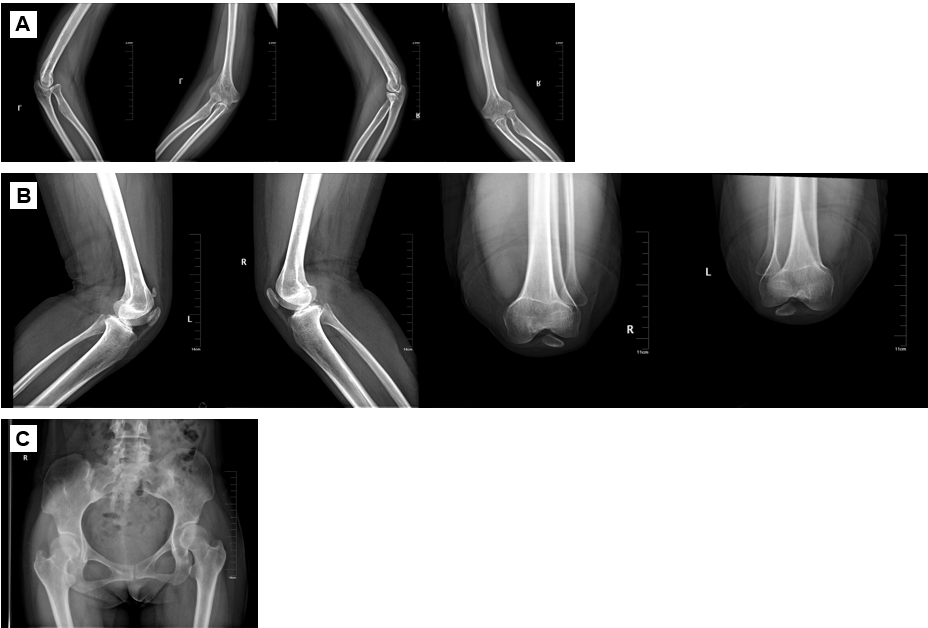
Fig S2. Radiographic findings of the proband’s mother. A. Elbow X-ray demonstrates right radial head subluxation and left radial head dislocation. B. Knee X-ray reveals hypoplastic patella without patellar dislocation. C. Pelvic X-ray shows right iliac horn and bilateral hip dysplasia.

| **Examination** | **Proband** | **Mother** | **Sister** |
| --- | --- | --- | --- |
| Urinary protein | Negative | Negative | Negative |
| Blood Creatinine (μmol/L) | 62.2 | 55.0 | 58.6 |
| Intraocular pressure (mmHg) | 17 | 19 | 14 |

Table S1: Urinary protein, blood Creatinine and intraocular pressure (non-contact tonometer method) result of affected members.
